# Treatments for Pulmonary Ricin Intoxication: Current Aspects and Future Prospects

**DOI:** 10.3390/toxins9100311

**Published:** 2017-10-03

**Authors:** Yoav Gal, Ohad Mazor, Reut Falach, Anita Sapoznikov, Chanoch Kronman, Tamar Sabo

**Affiliations:** 1 Department of Biochemistry and Molecular Genetics, Israel Institute for Biological Research, Ness-Ziona 76100, Israel; reutf@iibr.gov.il (R.F.); anitas@iibr.gov.il (A.S.); tamars@iibr.gov.il (T.S.); 2 Department of Infectious Diseases, Israel Institute for Biological Research, Ness-Ziona 76100, Israel; ohadm@iibr.gov.il

**Keywords:** ricin, pulmonary intoxication, countermeasures, antitoxins, disease-modifying agents, anti-ricin small molecules

## Abstract

Ricin, a plant-derived toxin originating from the seeds of *Ricinus communis* (castor beans), is one of the most lethal toxins known, particularly if inhaled. Ricin is considered a potential biological threat agent due to its high availability and ease of production. The clinical manifestation of pulmonary ricin intoxication in animal models is closely related to acute respiratory distress syndrome (ARDS), which involves pulmonary proinflammatory cytokine upregulation, massive neutrophil infiltration and severe edema. Currently, the only post-exposure measure that is effective against pulmonary ricinosis at clinically relevant time-points following intoxication in pre-clinical studies is passive immunization with anti-ricin neutralizing antibodies. The efficacy of this antitoxin treatment depends on antibody affinity and the time of treatment initiation within a limited therapeutic time window. Small-molecule compounds that interfere directly with the toxin or inhibit its intracellular trafficking may also be beneficial against ricinosis. Another approach relies on the co-administration of antitoxin antibodies with immunomodulatory drugs, thereby neutralizing the toxin while attenuating lung injury. Immunomodulators and other pharmacological-based treatment options should be tailored according to the particular pathogenesis pathways of pulmonary ricinosis. This review focuses on the current treatment options for pulmonary ricin intoxication using anti-ricin antibodies, disease-modifying countermeasures, anti-ricin small molecules and their various combinations.

## 1. Introduction

Ricin toxin, derived from the castor bean plant *Ricinus communis*, is a highly toxic protein that belongs to the type 2 ribosome-inactivating proteins (RIP) family [1]. Ricin binds to galactose residues at the cell surface via its lectinic B subunit (RTB) and then internalizes and traffics to the endoplasmic reticulum (ER), where the two subunits are reduced [2]. The catalytically active A subunit (RTA) translocates into the cytoplasm, where it depurinates a conserved adenine residue located in the 28S ribosomal RNA of the 60S subunit, thus leading to irreversible inhibition of protein synthesis and ultimately to cell death. Ricin is classified as a Category B agent by the U.S. Centers for Disease Control and Prevention (CDC) and is considered a potential bioterror agent mainly due to its high availability and ease of preparation.

Despite many efforts invested over the past decades, no clinically approved treatment against ricin poisoning has been established. To date, the only post-exposure measure found to be effective against pulmonary ricinosis in pre-clinical studies is passive immunization with anti-ricin neutralizing antibodies [3,4,5,6]. Recent data suggests that small-molecule compounds that either interfere with or inhibit the intracellular trafficking of ricin may also be beneficial against ricinosis [7,8,9,10]. Another approach has recently shown that the co-administration of antitoxin antibodies with immunomodulatory drugs enables toxin neutralization while decreasing lung injury severity, thus improving treatment outcomes [11,12].

In this review, we survey current treatment options for pulmonary ricin intoxication using anti-ricin antibodies, disease-modifying countermeasures, anti-ricin small molecule drugs or combinations of drugs-antitoxin. The rationale for screening additional drug candidates for combinatorial treatment is discussed, and suggestions for drugs that might be incorporated into future post-exposure therapy regimens are provided.

## 2. Ricin-Induced Cytotoxicity and Pathophysiology of Pulmonary Ricinosis

A better understanding of the mechanisms underlying both the cellular and physiological effects following pulmonary ricin poisoning are expected to assist in the development of novel treatment modalities against this type of exposure. Manipulation of these pathological processes will hopefully provide tools for clinical interventions that will attenuate lung damage and enhance therapeutic outcomes. In the following section, the current knowledge of the pathological and biochemical changes that occur following ricin intoxication is summarized.

### 2.1. Pathogenesis

Data regarding the pathological changes that occur following pulmonary ricin intoxication are mostly available from experiments performed in rodents, non-human primates and swine. The overall clinical picture is that the injury is mostly confined to the lungs and that the intoxicated animals suffer from marked interstitial pneumonia associated with massive neutrophil infiltration, perivascular and alveolar edema, fibrin deposition, hemorrhage and diffuse massive airway epithelial necrosis involving all lung lobes [13,14,15,16,17]. Eventually, flooding of the lungs leads to respiratory insufficiency and death. Recently, a swine model for pulmonary ricin intoxication was established by our group, allowing us to further assess the physiological and pathological changes that occur over time. It was found that the clinical manifestations comply with the accepted diagnostic criteria for acute respiratory distress syndrome (ARDS). As in other tested animal models, the pattern of local pro-inflammatory cytokine storming preceding massive neutrophil infiltration and increased vascular hyper-permeability was demonstrated [18].

### 2.2. Biomarkers and Cellular Stress Pathways

**Figure 1:**
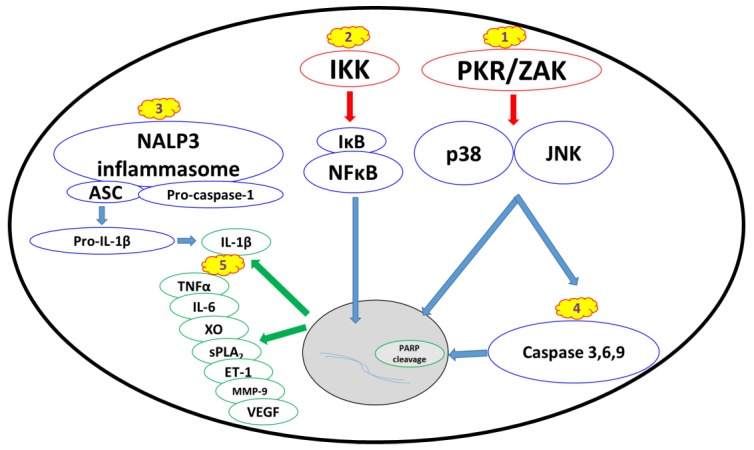
Ricin-induced activation of cell signaling pathways and downstream formation of damage mediators. (**1**) The ribotoxic stress response characterized by MAP3K (PKR and ZAK) activation of MAPK (p38 and JNK) signaling; (**2**) The nuclear factor kappa B pathway, which is activated upon IкK-induced IкB phosphorylation and degradation; (**3**) NALP3 inflammasome-mediated IL-1β activation; (**4**) Apoptotic cell death attributed to pro-apoptotic caspase activation; (**5**) Proinflammatory cytokines and damage mediators released upon activation of the various signal transduction pathways activated by ricin.

While the final outcome of ricin activity within the cell is the cessation of protein synthesis, it also leads to the activation of several cellular signaling pathways that in turn may activate multi-organ responses (Figure 1). These signals are further exacerbated by the inflammatory response and damage processes induced by the host, resulting in a “vicious cycle” of damage propagation.

#### 2.2.1. Ribotoxic Stress Response

Partial or complete ricin-mediated inactivation of ribosomes leads to the activation of a proinflammatory signaling pathway termed the “ribotoxic stress response.” Ricin can trigger the activation of JNK and p38 [19,20,21], which in turn increase the production of proinflammatory cytokines and apoptosis-mediated cell death [8,20,22,23,24]. The claim that MAP3K ZAK serves as an upstream activator of the ribotoxic stress response [23] is supported by the inability of ricin to activate p38 and JNK in ZAK-knockout macrophages in vitro and the lower ricin-induced pathology score following oral exposure in ZAK-knockout mice [25]. Another critical upstream mediator of the ribotoxic stress response is ribosome-associated RNA-dependent protein kinase (PKR) (Figure 1) [26], which also induces the activation of JNK and p38, as well as other signaling factors [27,28,29].

#### 2.2.2. Nuclear Factor Kappa B Pathway

An additional central downstream signaling pathway in the ricin-elicited stress response is the activation of nuclear factor kappa B (NFкB) (Figure 1). NFкB translocates to the nucleus upon IкB kinase (IкK)-induced IкB degradation and transactivates proinflammatory genes [30,31,32]. Activation of this signaling pathway is associated with many types of sterile lung injuries [33,34,35,36] and specifically with pulmonary ricinosis, where nuclear localization of NFкB was detected in mice intratracheally exposed to ricin [37]. Ricin was also shown to activate NFкB in pulmonary epithelial cell cultures [38].

#### 2.2.3. Proinflammatory Cytokines and Damage Mediators

As mentioned above, one of the hallmarks of pulmonary exposure to ricin is the activation of a massive inflammatory response in the lungs. The NALP3 inflammasome (also known as the NLRP3 inflammasome) promotes the cleavage of pro-IL-1β to active IL-1β by caspase-1 (Figure 1) and has a major impact on neutrophil infiltration and exacerbation of the overall inflammatory-mediated damage. It has previously been demonstrated [39] that inflammation— in particular pulmonary neutrophil infiltration and ensuing edema formation—is initiated by macrophage-dependent IL-1β signaling in mice exposed intratracheally to ricin and that ricin-induced IL-1β secretion from macrophages is dependent on NALP3 inflammasome activity. This primary event of IL-1β production is critical for the development of lung injury because the depletion of this cytokine significantly attenuates inflammation, as well as neutrophil pulmonary infiltration.

In addition to the early macrophage-dependent production of IL-1β in the lung tissue, various other pro-inflammatory cytokines and damage mediators (Figure 1) have been detected. Our laboratory has previously demonstrated an early and transient secretion of TNFα into the broncho-alveolar fluid (BALF) of pulmonary ricin-intoxicated mice [11]. TNFα is a major mediator of neutrophil-dependent vascular hyperpermeability [40], which plays a key role in lung pathologies, including ARDS [41].

Other cytokines and chemokines have also been detected in the BALF of ricin-intoxicated mice. For example, a rapid and significant rise in IL-6 levels was discerned as early as 6 h post-exposure [11]. This cytokine, identified as an early biomarker of acute lung injury and a predictive marker of morbidity and mortality, acts as a major proinflammatory mediator for the induction of an acute-phase response leading to a wide range of effects, including leukocyte recruitment and activation [42,43,44,45].

In addition to the proinflammatory cytokines described above, diverse damage mediators, such as secretory phospholipase A2 (sPLA_2_), vascular endothelial growth factor (VEGF), matrix-metalloproteinase-9 (MMP-9) and xanthine oxidase (XO), were detected in the BALF of mice following intranasal ricin intoxication [11]. The levels of the lipolytic enzyme sPLA_2_, a potent mediator of inflammation via hydrolysis and degradation of surfactant phospholipids [46,47,48], are significantly elevated following ricin intoxication. In rodent acute lung injury models, sPLA_2_ was found to promote neutrophil infiltration and edema formation [49,50].

An altered lung fluid balance, leading to increased permeability pulmonary edema, is a major pathophysiological characteristic of intranasal ricin intoxication. VEGF, which promotes vascular permeability and interstitial edema [51], is significantly increased following ricin intoxication. Consistent with this finding, the levels of the serum-resident enzyme cholinesterase, as well as total protein, are increased significantly in the BALF of intoxicated mice, indicating that the blood-lung barrier is severely impaired. Increased Evans Blue dye extravasation, as a marker for increased vascular permeability, has also been reported [12,39].

Consistent with these findings, the levels of the gelatinolytic enzyme MMP-9 rapidly increase after ricin intoxication, displaying a peak level equivalent to a >100-fold increase as soon as 24 h post-exposure. MMP-9 plays an important role in lung injuries [52] and correlates with alveolar-capillary permeability [53].

Increased levels of XO, an enzyme associated with oxidative damage and endothelial dysfunction-mediated edema formation [54], were measured after ricin intoxication and displayed a three-fold increase over control levels at 48 h post-exposure, followed by a ~10-fold increase over the next 24 h. The expression of XO, which results in localized formation of reactive oxygen species (ROS), was found to correlate with the severity of lung damage [55,56]. Ricin-induced oxidative stress has been reported in vivo following systemic intoxication of mice [57,58,59,60], and it has been suggested that NALP3 activation promotes ROS generation, which indirectly activates the inflammasome [61]. Hence, XO-derived ROS may also enhance inflammation via this route. Furthermore, ROS have been reported to mediate ricin-induced apoptotic cell death [62], a pathological process that will be further discussed below.

We have recently observed a significant increase in vasoconstrictor peptide endothelin-1 (ET-1) in the BALF of ricin-intoxicated pigs [18]. ET-1, the most abundant isoform of the endothelin peptide family, is produced by a variety of cells, including the airway epithelium and alveolar epithelial cells [63,64,65], and is known to be released in response to various pathological states [66,67]. Elevated levels of circulating ET-1 are considered a marker for endothelial dysfunction [68] and also correlate with increased pulmonary water contents [69]. Recent studies have suggested that ET-1 not only induces edema accumulation but also prevents edema resolution by impairing alveolar fluid clearance [64,70].

#### 2.2.4. Apoptosis and Changes in Cell Morphology

The induction of an apoptotic response following ricin intoxication (Figure 1) has been demonstrated in several cell types, including epithelial cells [16], endothelial cells [71,72,73] and macrophages [22,74,75]. This effect is associated with caspase-3 and PARP cleavage, which might be counteracted by the anti-apoptotic protein Bcl-2 [76,77]. It has also been suggested that ricin-induced apoptosis is mediated by p38 activation and is associated with TNFα production [22]. A single in vitro study demonstrated that the redistribution of intracellular zinc ions occurs early during ricin-induced apoptosis and that the exogenous addition of zinc ions may reduce apoptosis by inhibiting caspase 3, 6 and 9 activation, without affecting protein synthesis inhibition [24]. In addition to apoptotic events *per se*, ricin stimulates very rapid and dramatic morphological changes in primary endothelial cells, including the rounding of cells and formation of intercellular gaps, resulting in the passage of molecules through cell monolayers in vitro. These changes precede protein synthesis inhibition and may explain the vascular leak syndrome, which is associated with systemic ricin intoxication [78]. The enhanced sensitivity of endothelial cells may also explain ricin-induced vascular hyper-permeability in the setting of pulmonary exposure, similar to the vascular leak syndrome developed following systemic exposure.

## 3. Countermeasures for Ricin Intoxication

To date, there are no clinically approved post-exposure medical countermeasures against ricin intoxication. Pre-clinical studies indicate that anti-ricin small molecules may confer protection against pulmonary ricinosis, but only if administered before or shortly after intoxication. To obtain significant surviving ratios at clinically relevant time points following intoxication, the only effective countermeasure, to the best of our knowledge, is anti-ricin antibody-based therapy (“antitoxin”). The combination of antitoxin with small molecules that are anti-ricin targeted, or with compounds that attenuate pathological outcomes (“disease-modifying agents”) may improve protection in comparison to antitoxin treatment alone.

The next part of this review summarizes the current knowledge regarding the following potential countermeasures for ricin intoxication: (i) antitoxins; (ii) disease-modifying agents and (iii) small molecules. While some of the reviewed studies use systemic models of ricin intoxication, it is reasonable to assume that these therapies would also be beneficial, to some extent, during the course of pulmonary ricinosis.

### 3.1. Antitoxins

Over the years, various polyclonal and monoclonal ricin-neutralizing antibodies exhibiting a range of protection efficiencies have been described as post-exposure measures. The ricin molecule participates in many protein-carbohydrate and protein-protein interactions during the intoxication process. Accordingly, antibodies that effectively block the different binding steps have been found. For example, antibodies directed against the B-subunit of the toxin molecule can block the attachment of the toxin to the cell surface and thus inhibit its ability to enter the cell [4,79]. Antibodies directed against other epitopes located either on the A- or the B- subunit of ricin can interfere with its ability to interact with proteins involved in the retrograde transport of RTA into the cytosol [4,79,80]. Antibodies directed against the A-subunit of the toxin inhibit the catalytic activity of ricin in vitro [10,80,81,82] and are also found effective in vivo [5,6]; however, because these antibodies may dissociate from RTA during the retrograde transport process, it is highly possible that other mechanisms are responsible for the neutralizing activity of these antibodies.

From a clinical perspective, an extended therapeutic time window may be required for efficient treatment, because under various scenarios, therapeutic intervention may be implemented only after the passage of a considerable span of time following exposure. Taking into account the existing assays for the reliable and sensitive detection of ricin in a variety of samples, it can be reasoned that the trigger to treat, namely, the identification of ricin as the cause of intoxication, will be in the range of 24–48 h post-exposure. Unfortunately, the protection efficiencies of most of the reported antibodies decline sharply if they are not applied within several hours of exposure [11,83,84]. Table 1 enlists the monoclonal antibodies that were shown to elicit post-exposure protection. Importantly, extensive efforts are being made to improve the efficacy of the antibody [5] and to shorten the identification time in clinical samples. In this respect, we recently developed a unique method to detect active ricin in clinical samples. This method enables identification of the toxin in samples from pulmonary-intoxicated mice at early time points such as 3 h following intoxication [85]. In another set of experiments, we also demonstrated as a proof-of-concept that a combined treatment of anti-ricin antibodies with diverse drugs (“add-on therapy”) improved the survival outcome of intoxicated animals when treatment was initiated at 24 h post-exposure (described in detail below). In the following sections, other appealing possibilities of drugs that may be synergistically combined with anti-ricin antibodies and improve the treatment outcome are discussed.

### 3.2. Disease-Modifying Countermeasures

At later times following pulmonary ricin exposure, the pathophysiological condition of the intoxicated animals may have deteriorated due to the concomitant activation of several stress pathways, which exacerbate the pathological outcome. Accordingly, it can be hypothesized that by mitigating the activation of these pathways, a more favorable outcome will be attained. Such disease-modifying countermeasures can target the stress pathways described above, i.e., proinflammatory cytokines, pathologic damage mediators, inflammasome activation, stress-activated signaling pathways, apoptosis and many others (Table 2).

#### 3.2.1. Attenuation of Proinflammatory Cytokines and Damage Mediators

As mentioned above, the proinflammatory cytokines IL-1β, TNFα and IL-6 are elevated in the lungs of all animal models in which they are tested. Clinically approved drugs are frequently used to target these cytokines in many inflammatory-related pathologies [93]. Therefore, it is highly reasonable to apply anti-cytokine treatment in the course of pulmonary ricinosis. IL-1β and TNFα are early-formed cytokines; therefore anti-IL-1β or anti-TNFα drugs should be administered as soon as possible following intoxication, even if a co-administrated antitoxin is administered at a later time point. Supporting this notion, the clinically approved IL-1R antagonist (IL-1Ra) anakinra, a competitive inhibitor interfering with binding of IL-1α and IL-1β to their related receptors, attenuated lung injury in mice intratracheally intoxicated with ricin [39]. Consequently, this treatment induced a significant attenuation of cytokine storming and neutrophil recruitment, an improved histological score and extension of the mean time to death of ricin-intoxicated mice [39].

Inhibitors of other proinflammatory cytokines, such as TNFα and IL-6, have not yet been tested in ricin-intoxicated animals. Several anti-TNFα drugs are available clinically and can be chosen according to the species or effect requested (chimeric vs. humanized anti-TNFα, among others). Regarding IL-6, tocilizumab is a clinically approved anti-IL-6 drug, and additional anti-IL-6 agents are under clinical development [94], yet the proper animal model should be chosen carefully because tocilizumab does not cross-react with murine-IL-6 [95]. While most studies suggest that there is a positive correlation between the severity of the pulmonary damage and IL-6 levels following ricin intoxication, it should be noted that in other models of pulmonary damage, IL-6 attenuates lung injury [96,97].

Inhibitors of the enzymes involved in lung tissue degradation following ricin intoxication, namely sPLA_2_, MMP-9 and XO, which are markers of lipolytic, proteolytic, and oxidant activities, respectively, should also be tested. Although there are no approved specific inhibitors for MMP-9, doxycycline, as well as other tetracyclines were shown to directly interact with this enzyme, thus inhibiting its activity [98]. Several inhibitors of sPLA_2_ and XO are clinically available for diverse pathological indications. For example, mepacrine, an anti-malarial drug [99], is a potent inhibitor of sPLA_2_, demonstrating anti-inflammatory activities in lung pathologies. Specifically, mepacrine attenuated pulmonary vascular leakage following the intratracheal instillation of IL-1 [49,100], which, as stated above, is an important early mediator of murine pulmonary ricinosis [39]. The clinically approved XO inhibitor allopurinol significantly attenuated edema and improved the histological score in ventilator-induced lung injury [101]. In the setting of bleomycin-induced lung injury in mice, allopurinol reduced both pulmonary neutrophil infiltration and IL-1β levels [102]. Febuxostat, a non-purinic XO inhibitor, protected rats from LPS-induced lung injury, as reflected by decreased oxidative stress markers and reduced TNFα levels [103]. Furthermore, febuxostat significantly attenuated pulmonary neutrophil infiltration in acid-induced acute lung injury in mice [104].

XO is a representative marker of oxidative stress-induced damage. However, experimental evidence supports the notion that oxidants and oxidative stress are strongly associated with acute lung injury, having many more potential sources of ROS. These ROS may lead to direct DNA damage, lipid peroxidation, protein oxidation and proinflammatory gene upregulation [105]. There are many antioxidants used clinically, including N-acetylcysteine (NAC)—a widely used mucus-dissolving over-the-counter medication—which was shown to suppress the release of IL-1β from bone marrow-derived macrophages incubated with ricin [21]. Epigallocatechin gallate (EGCG), an antioxidant found in green tea, diminished ricin-induced cytotoxicity in cell cultures [106,107]. Several antioxidants, such as butylated hydroxyanisole (BHA) and vitamin E succinate, were also shown to provide protective effects in vivo against systemic ricin intoxication [58]. Although the pathological outcomes of systemic ricin exposure are considerably different from those following pulmonary exposure, it is worthwhile to evaluate the above-mentioned antioxidants in a pulmonary ricinosis model because the efficacy of these compounds have also been demonstrated in various types of lung pathologies. For example, vitamin E effectively reduced the oxidative burst and neutrophil pulmonary infiltration in endotoxin-induced lung injury in mice [108]. EGCG attenuated LPS-induced lung injury in mice by reducing neutrophil accumulation, edema formation and pulmonary damage severity [109]. In another study, EGCG was found to reduce seawater aspiration-induced acute lung injury in rats via the regulation of inflammatory cytokines [110].

The elevated levels of VEGF following intranasal ricin intoxication [11] are closely related to pulmonary vascular hyperpermeability and edema formation [51,111], the ultimate cause of respiratory failure and death following pulmonary ricinosis. There are various clinically approved drugs antagonizing the effect of VEGF, i.e., bevacizumab (Avastin) [112] and aflibercept [113], which can be used in the setting of pulmonary ricinosis. Yet, it should be noted that the presence of VEGF in the alveolar space could be protective against diverse settings of murine lung injury [114]; therefore, an anti-VEGF regimen should be assessed carefully. The elevated levels of ET-1 measured in pig BALF following intratracheal ricin intoxication [18] may also be associated with edema formation and progression. Several ET-1 antagonists are clinically available, for example, bosentan [115] and tezosentan [116], which should be evaluated for their efficacy in pulmonary ricin intoxication.

In addition to ET-1 and VEGF, many other pathological effectors involved in the process of edema can also be targeted using a large repertoire of clinically approved and preclinically tested drugs. For example, iloprost improves endothelial barrier function in a murine model of LPS-induced lung injury [117]. Enhancing vascular endothelial barrier integrity with the Tie2-agonist Vasculotide, a patented molecule under experimental investigation, improved survival in murine models of influenza even when administered as late as 72 h following infection [118]. β-agonists, e.g., salbutamol, which is frequently used for the treatment of asthma and other pulmonary pathologies, may accelerate the clearance of extravascular lung water and promote anti-inflammatory effects [119,120,121].

An additional disease-modifying approach is to use wide-range anti-inflammatory drugs or immunomodulators for the treatment of pulmonary ricinosis. In that respect, we have previously shown that doxycycline [11] and ciprofloxacin [12], antibacterial agents repurposed as immunomodulators, significantly attenuated various proinflammatory markers, such as IL-1β, IL-6, XO, neutrophil lung count and edema markers. These highly effective drugs are widely available and are not expensive, rendering them attractive as anti-ricin therapies. Supporting the notion that the coverage of as many damage mediators in parallel would achieve a better impact, it was demonstrated in several non-ricin-mediated lung pathologies that a combinational treatment of two drugs obtained a better outcome than each drug alone. For example, a combinatory treatment with vitamin E and the corticosteroid dexamethasone resulted in a sharper decrease in BALF neutrophil content and less severe oxidative damage following endotoxin-induced lung injury than each drug alone [108]. Similarly, the co-administration of NAC with steroids and deferoxamine improved the outcome of chlorine-induced [122] or LPS-induced [123] acute lung injury in mice and rats.

#### 3.2.2. Ribotoxic Stress Response Inhibitors

It has previously been demonstrated that the inhibition of p38 protects cells from ricin-induced effects [22]; however, p38 inhibitors are often toxic and therefore exhibit very limited success in clinical trials. Recently, using computer-aided drug design, UM101, a novel inhibitor of p38α, the major isoform responsible for the proinflammatory effects of this MAPK, was evaluated [124]. This compound was more potent than the non-selective p38 inhibitor SB203580 in stabilizing endothelial barrier function and reducing inflammation in the course of LPS-induced murine acute lung injury. In a high-throughput cell-based assay, one of the few identified compounds, PW66, was found to diminish the ricin-induced ribotoxic stress response by interfering with the activation of p38 and JNK as well as inhibiting TNFα secretion [8]. The status of current JNK inhibitors was recently reviewed by Cicenas [125], and it is worth mentioning that several JNK inhibitors, particularly SP600125, exhibit protective effects against acute lung injury in vivo [126,127,128,129].

The MAP3Ks ZAK and PKR, which are upstream activators of p38 and JNK, may also serve as targets for the treatment of pulmonary ricinosis. Indeed, sorafenib and nilotinib, which are clinically approved inhibitors with a high affinity for ZAK, decreased the ricin-induced ribotoxic stress response (activation of both p38 and JNK) in macrophages [21]. The ZAK inhibitor DHP-2 decreased the p38 and JNK-induced ribotoxic stress response in epithelial cells, increased cell viability and further decreased caspase-3 activation and proinflammatory gene transcription following incubation with ricin [23]. Small molecule inhibitors of PKR were also identified [130] and were found to be effective both in vitro and in vivo. For example, 2-AP and C16 suppressed the production of the proinflammatory cytokine IL-8 in monocytes [131], whereas PKRi was effective in a mouse model of long-term memory [132]. In addition, the PKR inhibitor imoxine, as well as 2-AP, improved glucose hemostasis and performed anti-inflammatory activities in an obese diabetic murine model [133].

#### 3.2.3. NFкB Inhibitors

Activation of the NFкB signaling pathway induces the transactivation of proinflammatory genes and plays a major role in ricin-mediated pathogenesis. A screen of 2800 clinically approved drugs and bioactive compounds was conducted to identify novel NFкB inhibitors [134], of which 19 exhibited very high potency, including bortezomib, daunorubicin, digitoxin, emetine, sorafenib, sunitinib, tioconazole and zafirlukast. Additional NFкB-inhibiting drug candidates, including IкB kinase inhibitors, are discussed elsewhere [135,136]. Importantly, some NFкB inhibitors were found to be effective in animal models of pulmonary inflammation, such as Bayer’s ‘Compound A,’ which demonstrated broad anti-inflammatory activity in mice and rats following intraperitoneal administration of LPS [137]. In addition, using the BMS-345541 IкK inhibitor, the levels of lung-activated NFкB, proinflammatory cytokine levels, neutrophil influx and edema formation were all reduced in LPS-challenged mice [138].

#### 3.2.4. NALP3 Inflammasome Inhibitors

In addition to the direct induction of the NALP3 Inflammasome by ricin [21,39], the extracellular release of endogenous molecules such as uric acid [102], ATP [139] and neutrophil-derived extracellular histones [140], may stimulate inflammasome activation following ricin-mediated cell necrosis. This highly efficient activation of the NALP3 inflammasome by both ricin and the proinflammatory mediators released by dying cells may be a target for a potential therapeutic intervention. Several NALP3 inhibitors, including parthenolide, glyburide (a clinically approved anti-diabetic drug), 5-chloro-2-methoxy-*N*-[2-(4-sulfamoylphenyl) ethyl]benzamide and isoliquiritigenen (a chemical compound found in licorice) [141,142,143,144], are currently under investigation. The small molecule MCC950 was recently suggested as a therapy for NALP3-associated syndrome, exhibiting a specific and highly potent inhibitory activity that resulted in reduced IL-1β production in mice [145]. Likewise, the ketone metabolite β-hydroxybutyrate (BHB) reduced NALP3 inflammasome-induced IL-1β and IL-18 production [146]. Additional inflammasome inhibitors have been extensively reviewed by Shao [147]. These include clinically approved non-selective inhibitors (interferons-α/β), autophagy-enhancing agents (resveratrol, arglabin and HU-308, which is a representative cannabinoid receptor 2 agonists), and microRNAs (microRNA-223).

#### 3.2.5. Compounds Counteracting Apoptosis and Cell Morphology Changes

Ricin-induced apoptosis may be mediated by various stimuli, such as p38, TNFα [22], ROS [62] and zinc redistribution [24]. Accordingly, there are many options to reduce apoptosis, including the above-mentioned drugs that target these stimuli. In this respect, zinc deficiency aggravates several types of lung injury via NFкB activation, VEGF overexpression and alveolar epithelial/macrophage cell dysfunction [148,149,150]. The administration of zinc ions alleviates several types of lung injury in rodents [151,152] by decreasing the levels of XO, oxidative stress markers, lung neutrophil recruitment and NFкB activation. Importantly, a link between zinc, caspase-3 and adherent junction-related cell-to-cell contact was demonstrated. Zinc deprivation in the presence of pro-apoptotic stimuli accelerated caspase-3 activation and E-cadherin and β-cathenin proteolysis and induced an increase in paracellular leak across epithelial cell monolayers. Zinc supplementation, but not caspase inhibition, protected the lung epithelial barrier [153]. Consequently, it might be hypothesized that zinc also inhibits changes in cell morphology that might be associated with the vascular leak syndrome [78].

Reducing the progression of apoptotic-mediated cell death may also be achieved by interfering with the apoptotic machinery using clinically approved drugs or compounds that are under clinical investigation [154,155]. For example, the compound PW69 was demonstrated as a ricin-induced caspase 3/7-mediated apoptosis suppressor [8]. Excitingly, a recent work revealed that the antiparasitic drug bithionol potently inhibited caspases- 3/7, 6, and 9 and exhibited pronounced protection against ricin-induced cell death [156].

### 3.3. Anti-ricin Small Molecules

**Figure 2:**
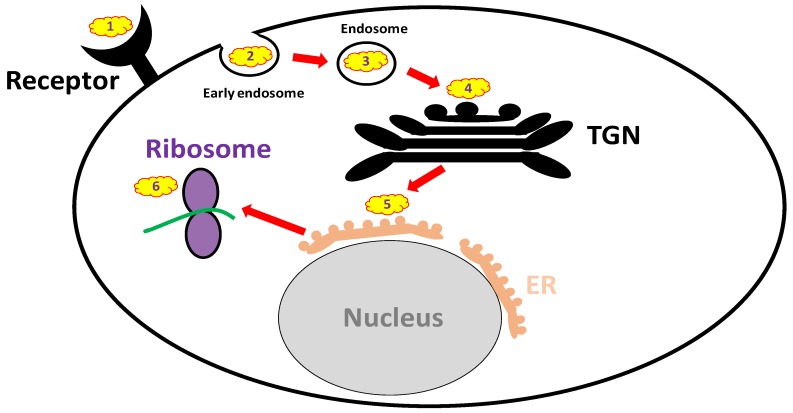
Cellular targets for anti-ricin small molecule compound-based treatment. (**1**) Receptor mimicry; (**2**) Blockers of endocytosis; (**3**–**5**) Retrograde trafficking blockers; (**6**) Active-site inhibitors. TGN: trans-Golgi network; ER: endoplasmic reticulum.

Anti-ricin antibodies are expected to be non-active when administered after the toxin has already entered the cell, while small molecules that can penetrate the cells might be effective at that time point. Over recent years, an extensive search was held for small molecule inhibitors of ricin. High-content screens have revealed attractive, potentially effective compounds that may be used therapeutically; however, all are still under preliminary investigation. The mechanisms of action of these small molecules are diverse, targeting different cellular pathways of ricin, e.g., membrane binding, intracellular trafficking and the active site (Figure 2 and Table 3). Importantly, there are some limitations and prerequisites for such small-molecule-drug candidates, including safety issues concerning their clinical use. Additionally, they should preferably be water soluble and well absorbed from the GI tract if taken orally. Drug candidates aimed at targeting the active site or inhibiting intracellular trafficking are required to penetrate cell membranes as well.

It is a great advantage if the drugs are already clinically approved for other indications (repurposed drugs) and also commonly available and not expensive. A list of candidate anti-ricin small molecule drugs categorized by their mode of actions is provided below.

#### 3.3.1. Receptor Mimicry (RTB Binders)

Asialofetuin (ASF), which contains 12 terminal galactose residues per molecule [157,158] and binds ricin 1000 times better than monovalent galactose [159], is an excellent in vitro scavenger of ricin. However, it is rapidly cleared from the circulation and therefore cannot be used clinically. Extensive work has been performed to obtain better receptor mimicry. Glycosphingolipid (GSL)-, lactose-, and galactose-based derivatives (Table 3) were found to be potentially good candidates for this manner. The gangliosides GM1 and GM3 protect cells from ricin-induced intoxication [160], while the synthetic GSL analogues beta-lactosyl-ceramide, beta-d-galactosyl ceramide, asialo-GM1 and serum albumin-based neoglycoconjugates were shown to be selective and potent ricin binders in vitro [161,162]. Lactose-based glycopolymers were found to be effective for capturing ricin in a cell-free system, as well as for inhibiting cell binding [163,164]. In a different experimental setting, a synthetic galactose-based surfactant efficiently sequestered ricin from aqueous solution, but due to its water-insolubility, it must be formulized prior to its application for ricin intoxication therapy [165]. A galactose-based biantennary oligosaccharide effectively bound to ricin in a cell-free system [166], whereas a chemically modified glycoprotein containing triantennary N-linked oligosaccharides reduced the cytotoxicity of ricin more than 1000-fold in cultured cells [167]. Additional studies were performed with the closely related protein *Ricinus communis* agglutinin (RCA), demonstrating potent binding to Galβ1-4GlcNAc, with specificity for highly branched glycans containing this structure [168]. EGCG, a potent antioxidant possessing anti-inflammatory properties [109,110], was also suggested to interfere with the binding of RTB to lactose-conjugated sepharose [107].

Although all of these molecules effectively antagonize ricin in vitro or in cell free systems, to our knowledge, there are no data available regarding the in vivo efficacy of anti-ricin receptor mimetic-based small molecules.

#### 3.3.2. Endocytosis Blockers

Research conducted decades ago revealed that the co-incubation of an inhibitor of glycolysis (2-deoxyglucose) and an uncoupler of oxidative phosphorylation (sodium azide, NaN3) potently inhibits ricin endocytosis and protects cells against intoxication, indicating that endocytosis is a critical step in ricin cellular entry [169]. Later work demonstrated that cytochalasin D and the clinically approved drug colchicine selectively inhibit the endocytic uptake of ricin from non-clathrin-coated areas of cell membranes. Furthermore, colchicine reduces the catalytic activity of ricin (protein synthesis arrest) in cell culture [170].

#### 3.3.3. Trafficking Blockers

After internalization into the cells, ricin is transported from early endosomes to the ER via the Golgi apparatus, an entrance pathway termed the “retrograde trafficking route.” Several molecules were found to block ricin translocation to the cytosol, e.g., brefeldin A (BFA) [171], 3′-azido-3′-deoxythimidine [172] and mansonone-D [173]. BFA, a fungal antibiotic, which inhibits anterograde vesicular transport by disrupting the Golgi apparatus, is considered to be the first small molecule identified that protects cells from ricin [171]. However, whereas BFA protects cells from the cytotoxicity induced by ricin, it may under some circumstances enhance ricin toxicity in other cell lines [174,175]. In addition, it was recently demonstrated that benzyl alcohol, which is widely used as a food and medical preservative, inhibits ricin membrane trafficking between endosomes and the trans-Golgi network, thus providing protection against ricin-induced cytotoxicity [176].

In the past decade, several high-throughput screens were conducted, including a high-content screen of ~3000 compounds that identified several small molecule candidates that interfered in vitro with the retrograde translocation of ricin or stabilized RTA in the ER [177]. With these screens, the greatest progress in the field of ricin trafficking blockers was recently achieved. Small molecules that selectively block retrograde trafficking at the early endosome/trans Golgi network interface were identified. These highly selective, non-toxic molecules were efficient against pulmonary ricinosis in mice, especially Retro-2 administered prophylactically. This molecule was found to be highly potent, exhibiting bioactivity in the nanomolar range [178]. In a different experimental setting, characterization of a common pharmacophore of retrograde trafficking inhibitors, such as Retro-2 and its achiral analog DA2MT, offered new insights into lead compound identification and optimization for ricin and other RIP antidote development [179]. Additional inhibitors of cellular trafficking are discussed elsewhere [180], and some of the molecules may be potentially effective if proven safe when used against ricin intoxication.

In addition to the trafficking inhibitors mentioned above, Bassik et al. demonstrated that ricin trafficking to the ER was effectively blocked in vitro upon hydroxymethylglutharyl (HMG)–CoA reductase inhibition with atorvastatin, a popular cholesterol-lowering drug [181].

#### 3.3.4. Reductive Activation Inhibitors

A reduction-dependent disassociation of the RTA-RTB inter-subunit disulfide bond is required for the intracellular activation of ricin, namely, the translocation of RTA from the ER to its target site, the cytosol. Several enzymes responsible for this process have been identified, e.g., protein disulfide isomerase (PDI), thioredoxin reductase [182], glutathione disulfide oxidoreductase [183] and TMX, a transmembrane thioredoxin-related protein member of the PDI family [184]. Among these enzymes, thioredoxin reductase and PDI may be inhibited by the clinically approved drugs, auranofin (used therapeutically for rheumatoid arthritis, [185]) and the antibacterial agent bacitracin [186], respectively. Indeed, auranofin significantly inhibits ricin-mediated cytotoxicity [182].

#### 3.3.5. Active Site and RTA Inhibitors

The search for RTA active site inhibitors began decades ago and included purine-based inhibitors and pterin-like and single ring pyrimidine-based derivatives, as extensively discussed elsewhere [10]. It should be mentioned, however, that many of these compounds are extremely insoluble and/or toxic to cells. According to a single publication in the literature, the clinically approved polyamine difluoromethylornithine, a positively charged compound, which was proposed to bind ribosomes and RNA, prolonged the time to death in mice intraperitoneally intoxicated with ricin, likely by blocking the active site of the toxin [58]. In addition, in a recent screening of ~80,000 compounds in vitro, 20 molecules with significant anti-ricin activity were identified, one of which (4-fluorophenyl methyl 2-(furan-2-yl)quinolone-4-carboxylate) exhibited significant therapeutic activity [9]. A computer modeling identified this compound as a ricin active site blocker. More specific active site blockers targeting the ribosome inactivating protein (RIP)-α-sarcin/ricin loop (SRL) interface were found to be effective, conferring up to 20% cell protection against ricin at nanomolar concentrations [187]. In addition, an RTA-ligand RNA aptamer-based approach demonstrated partial protection in a cell-based cytotoxicity assay [188], supporting the potential use of anti-RTA aptamers as ricin inhibitors.

The identification of new classes of RTA inhibitors by virtual screening was also conducted. Two compounds (out of 50,000) showed modest to strong ricin inhibition in a cell-based assay, which was, however, accompanied by some cytotoxicity [189]. In a recent study [190], baicalin, extracted from the plant *Scutellaria baicalensis* and used as a Chinese medical herb, reduced ricin-mediated cytotoxicity in vitro and conferred significant post-exposure protection in mice intraperitoneally exposed to ricin. Baicalin is an RTA inhibitor with a novel mechanism of action. Rather than occupying the active site, it induces toxin oligomerization upon extensive hydrogen bond networking formation with RTA. The potential protective effects of baicalin in pulmonary ricinosis remain to be evaluated.

### 3.4. Multiple Pathway-Interfering Anti-Ricin Agents

As mentioned earlier, auranofin inhibits the reductive activation of ricin by thioredoxin reductase [182], yet it may also decrease NFкB activation via IкK inhibition [191]. Notably, auranofin should be cautiously used since thioredoxin reductase inhibition is often associated with a concomitant increase in ROS formation [192]. Baicalin, a direct anti-ricin agent that forms oligomers through interactions with the RTA, may also function as an antioxidant [193,194] and an anti-inflammatory agent, as reflected by attenuation of LPS-induced lung injury in rats and mice [195,196,197]. In addition, resveratrol, an antioxidant [198] that may also act as a NALP3 inhibitor through autophagy enhancement [147], may have a protective effect against ricinosis. The actin depolymerizing agent cytochalasin D, which was reported to inhibit the uptake of ricin [170], may also inhibit NALP3-dependent IL-1β production [199,200]. Amifostine, a potent antioxidant [201], inhibited p53-dependent apoptosis by reducing apoptosis-related gene transcription [202,203]. Colchicine, a selective blocker of the cellular endocytic uptake of ricin [170], is also a microtubule-depolymerizing agent [204] that impairs the mobility and phagocytic activity of neutrophils. In particular colchicine was reported to attenuate phosgene-induced lung injury and to improve mouse survival by reducing neutrophil influx into the lungs even when given 30 min post exposure [205]. Atorvastatin, an HMG–CoA reductase demonstrated to operate as a retrograde trafficking blocker of ricin [181], as well as other HMG–CoA reductase inhibitors, were reported to attenuate non-ricin-mediated acute lung injury parameters, as reflected by reduced levels of proinflammatory cytokines and markers of edema [206,207,208].

### 3.5. Drug-Drug Combinational Treatment

Due to the multiplicity of ricin-induced damage pathways and effectors, an improved therapeutic outcome may be achieved upon combinational treatment of several drugs. Pathogenesis characterization of any treatment should assist tailoring the optimum additive countermeasure for efficient damage coverage. Clearly, there are many combinational treatment options, and therefore, the use of concomitant medication should be established wisely. For example, ZAK inhibition attenuates p38 and JNK activation in cell culture without any influence on NALP3 activation [21]. Therefore, if relevant to in vivo settings, ZAK inhibition should be combined with NALP3 inhibitors or anakinra following pulmonary ricin intoxication. As mentioned above, auranofin is a good candidate for ricin intoxication therapy due to its dual inhibition activity on both NFкB signaling and thioredoxin reductase. However, as mentioned earlier auranofin sensitizes cells to oxidative stress-mediated damage [192]. Accordingly, the co-administration of an antioxidative compound could improve treatment outcomes. NFкB knockdown in vitro [38] decreases the levels of several proinflammatory mediators but not IL-6. Hence, combined NFкB inhibition with anti-IL-6 medication is worth attempting. In the same experiment, incubating cells with anti-TNFα did not have any influence on NFкB activation, implying a beneficial effect of NFкB/TNFα combined inhibition, pending the relevance of this finding to pulmonary ricin intoxication in vivo. Miller et al. reported that some NFкB inhibitors promote caspase 3/7 activation [134]; therefore, targeting these effectors in parallel to NFкB inhibition may be advantageous. In addition to the aforementioned classical drug-drug combination, inhibition of these stress-related processes with the co-administration of anti-ricin small molecules, i.e., trafficking blockers may be useful.

### 3.6. Antitoxin-Drug Combinational Treatment

Administration of anti-ricin antibodies is less effective if applied at late time points following intoxication [83,84]. However, it was previously shown that the co-administration of antitoxin together with various anti-inflammatory drugs could significantly expand the therapeutic time window [11,12]. In the first study, the survival rates of mice treated with anti-ricin antibodies at 24 h post-pulmonary intoxication were considerably improved by the co-administration of doxycycline [11], an antibacterial agent with broad anti-inflammatory activity [209,210,211,212,213]. Doxycycline promoted a significant reduction of proinflammatory cytokines and damage mediators (IL-1β, IL-6, XO, VEGF and pulmonary ChE) in intoxicated mouse BALFs. Because doxycycline treatment *per se* did not confer protection, an “add on” effect obtained following antitoxin-doxycycline combination treatment was monitored. Indeed, doxycycline or antitoxin administration as monotherapies did not decrease the levels of BALF MMP-9; however, a combined drug-antitoxin treatment resulted in a significant reduction of this mediator [11]. The second efficient combinational drug-antitoxin-based therapy was achieved using ciprofloxacin as the co-administered drug. Ciprofloxacin is an antibacterial agent that also possesses potent immunomodulatory properties, a feature that is mainly associated with decreased synthesis of proinflammatory cytokines [214]. Co-administration of ciprofloxacin with antitoxin dramatically improved survival through effective attenuation of neutrophil infiltration and edema. These findings illustrated that ciprofloxacin led to a significantly decreased proinflammatory cytokine response.

Thus, in BALFs of ciprofloxacin-treated ricin-intoxicated mice, IL-6 levels decreased by ~90%, while a significant increase in levels of the anti-inflammatory cytokine IL-10 was observed. Pulmonary levels of the damage markers XO, protein, ChE and Evans Blue dye extravasation were also significantly attenuated upon ciprofloxacin treatment [12]. It appears that ricin, by virtue of being highly inflammatory, persistently stimulates acute inflammatory responses with which doxycycline or ciprofloxacin alone cannot cope. Antitoxin-based treatment is therefore required to halt any further propagation of proinflammatory signals by virtue of toxin neutralization, while the combined drugs exert their beneficial effects by dampening the inflammation-related assaults that have already developed.

Indeed, although treatment with the drugs alone reduced inflammation-related factors to a considerable extent, the values remained higher than in control mice. It appears, therefore, that keeping the inflammation at bay promotes the expansion of the therapeutic time window for antitoxin intervention. Supporting this argument, the time to death was delayed in IL-1-knockout mice compared with naïve mice following intratracheal ricin intoxication [39]. It is important to mention, that we have also examined the combination of antitoxin with dexamethasone, a highly potent corticosteroid, as it may be assumed that patients exhibiting an unknown inflammatory syndrome will be treated with these agents long before the specific cause is known. Surprisingly, although steroids possess broad anti-inflammatory activities and seem ideal for use in suppressing the ricin-induced inflammatory pathways, co-administration of dexamethasone did not confer any improvement in surviving ratios, unless given before, or shortly after the onset of intoxication. Under these circumstances we believe that non-steroidal-based immunomodulators are better candidates for combinational antitoxin-drug treatment. Nevertheless, we do believe that the usage of steroids in ricin-intoxicated victims, whether the treatment is given early, or at late times following exposure, should be beneficial. This argument is supported by the fact that treating mice with dexamethasone in combination with the antitoxin-doxycycline regime, conferred enhanced protection as compared to antitoxin-doxycycline alone [11].

Taken together, drug-antitoxin concomitant medication is emerging as the best approach for the treatment of pulmonary ricinosis, and the drugs and small-molecules that were reviewed above may help to improve this therapy.

## 4. Summary and Future Prospects

To date, the only applicable countermeasure for pulmonary ricinosis at clinically relevant time points following exposure is antitoxin administration. Small molecules inhibiting the intracellular trafficking of ricin (e.g., Retro-2) or interacting with RTA (baicalin) were shown to be efficient in vivo only when administered shortly after intoxication (the latter was not tested in pulmonary ricin exposure). Drug-antitoxin based therapy improves treatment outcome, however, the timing of each pharmacological intervention should be carefully chosen. For example, treatment with anti-IL-1β or anti-TNFα should be optimal when administrated at early times following intoxication, while immunomodulators and stress-activated pathways inhibitors may be beneficial also at later time points. Anti-ricin small molecules should be administered as soon as possible following intoxication.

To improve treatment outcomes, it is highly reasonable to simultaneously target several stress pathways, as well as cellular components related to the toxicity of ricin (binding, trafficking, catalytic activity etc.), as long as the additive toxicity of this combinational treatment does not pose a problem. Even small molecules that were not demonstrated to confer protection in vivo may be beneficial when included in an anti-ricin drug cocktail.

In addition to the countermeasures discussed in this review, several other therapeutic strategies should be considered following pulmonary ricinosis. Because this pathology complies with the clinical criteria for ARDS, pharmacologic treatments tested for this syndrome should be evaluated. As it is suggested that treatment against pulmonary ricin intoxication includes an antitoxin, drugs which were tested for ARDS and demonstrated only partial efficacy in clinical trials should be reconsidered as components of a combined drug-antitoxin treatment modality. The relevant pharmacological options include corticosteroids (as mentioned above), vasodilators, decreased alveolar surface tension, thromboxane synthase and 5-lipoxygenase inhibitors, antioxidants, immunonutrition, increased clearance of alveolar edema, enhanced repair of the alveolar epithelium, anticoagulants, hematopoietic colony-stimulating factors, and prevention of fibrosis, all of which have been extensively reviewed elsewhere [215,216,217,218].

Targeting the cytokine storm is also an attractive strategy to manipulate the overwhelming inflammation process that develops in the lungs following pulmonary ricin intoxication. Pharmacological strategies for this purpose were exhaustively discussed by D’Elia et al. and include stimulation of the cholinergic anti-inflammatory pathway, e.g., by GTS-21, which is a selective α7-acetylcholine (ACh) nicotinic receptor agonist found in clinical trials, and CNI-1495, an α7ACh agonist under pre-clinical testing. Prostaglandin-, cyclooxygenase- and platelet-activating factor- inhibitors, as well as chemokine manipulation, may also attenuate cytokine storm. The active resolution of tissue damage by resolvins, lipoxins and protectins is also relevant [219]. In particular, the activation of the cholinergic anti-inflammatory pathway following nicotine administration two hours after systemic ricin exposure significantly delayed and reduced mortality in mice [220], although the relevance to pulmonary ricinosis remains to be elucidated.

Neutrophils are considered a major hallmark of pulmonary ricinosis. Aggressive or prolonged neutrophil responses result in deleterious inflammatory conditions and tissue destruction. Potential new drug candidates to control of neutrophil activity were reviewed by Burgos et al. [135]. It is of great importance to follow the progression in this field and consider using agents counteracting neutrophil activity in the course of pulmonary ricinosis. Targeting p38 may be of a specific interest because it is involved in both neutrophil migration and chemotaxis in vivo [221] and in the ribotoxic stress response, as mentioned above. Comprehensive understanding of intravascular danger signals (e.g., formyl-peptide signals released from necrotic cells) that guide neutrophils to the site of sterile injury should be harnessed in an effort to attenuate neutrophil-derived injury [222].

Pharmacological interventions against systemic capillary leak syndrome were reviewed by Druey et al. [223]. Several clinically used drugs were offered to alleviate this syndrome, and these should be tested for their beneficial effects in pulmonary ricinosis.

Immune selective anti-inflammatory derivatives (ImSAIDs), based on salivary gland-derived peptides, should also be considered for evaluation in pulmonary ricin exposure. The tripeptide ImSAID Phe-Glu-Gly (FEG) attenuated both systemic [224] and pulmonary [225,226] inflammation. FEG significantly ameliorated endotoxin-induced lung injury in both a prophylactic and therapeutic manner in rats [227].

In addition, regenerative therapies should also be applied for the treatment of lung pathology following ricin intoxication. These therapies include the aforementioned resolving/lipoxin/protectin-based therapy, as well as keratinocyte growth factor (KGF)-mediated enhanced repair of the alveolar epithelium [216,228,229]. Cell therapy using a mesenchymal stromal cell-based approach, reviewed by Johnson et al., can also be performed, depending on its clinical progress [230].

Small molecules that interfere with the cell trafficking of ricin were comprehensively discussed in this review. Although considerable progress was achieved following the development of Retro-2, additional work should be performed to improve the effectiveness of trafficking blockers. Bassik et al. [181] used a mammalian genetic approach to reveal pathways underlying ricin susceptibility. Many intracellular factors, familiar as well as unexpected, were found to be involved in the toxicity of ricin. Knockdown of several of these factors was strongly protective, while knockdown of others, increased the sensitivity to ricin. Profound sensitization to ricin was found upon depletion of coat protein I (COPI) components, which are normally involved in retrograde transport. This is presumably due to the upregulation of compensatory alternative pathways or to the fact that COPI normally functions in transport steps that divert ricin from ER. Additionally, in contrast to all depleted ribosomal components, which sensitize cells to ricin, the knockdown of the ribosomal proteins RPS25 and ILF2/3, whom interact with RPS25 conferred ricin resistance. The depletion of the two poorly characterized genes *WDR11* and *C17orf75* sensitized cells to ricin. Specifically, *WDR11* was suggested to participate in autophagy-mediated ricin degradation. The clinical aspects of this work have not been elucidated, but it is reasonable to assume that targeting novel proteins that may be related to ricin susceptibility should be utilized in the future to develop novel anti-ricin therapeutic strategies.

In conclusion, anti-ricin post-exposure treatment should include the following: antitoxin, as an obligatory component, combined with (i) a disease modifying countermeasure (i.e., anti-inflammatory/immunomodulator agent); and/or (ii) a small molecule anti-ricin inhibitor (i.e., cell trafficking blocker). Pending clinical progression, the treatment at later stages may include regenerative therapies, gene- and cell-based treatments, and any other beneficial therapy that remains to be discovered. Optimum treatment should be tailored based on thorough pathological studies, specifically focusing on damage mediators that were not effectively attenuated following a treatment of choice.

## Figures and Tables

**Table 1:** Monoclonal antibodies shown to be protective against ricin when administered post-exposure.

Antibody Name	Antibody Type	Target	Reference
RAC18	murine; chimeric	RTA	[86]
PB10	chimeric		[87]
RA36	murine		[88]
43RCA-G1	humanized		[89]
GD12	murine; chimeric		[90]
MH1	chimeric		[6]
MH36	chimeric		[6]
JB4	chimeric	RTB	[87]
RB34	murine		[88]
RB37	murine		[88]
D9	murine; humanized		[91,92]
MH2	chimeric		[6]
MH73	chimeric		[6]
MH75	chimeric		[6]
MH77	chimeric		[6]

**Table 2:** Summary of disease-modifying countermeasures.

Pathway	Target	Inhibitors
Proinflammatory cytokines	IL-1β	anakinra, immunomodulators
	TNFα	anti-TNFα agents, immunomodulators
	IL-6	tocilizumab, immunomodulators
Damage mediators	XO	allopurinol, febuxostat, antioxidants
	sPLA2	Mepacrine
	ET-1	bosentan, tezosentan
	MMP-9	Doxycycline
	VEGF	bevacizumab, aflibercept
NFкB pathway	NFкB	NFкB inhibitors, ‘Compound A’
	IKK	IкK inhibitors, auranofin, BMS-345541
MAP3K	PKR	2-AP, C16, imoxine, PKRi
	ZAK	sorafenib, nilotinib, DHP-2
MAPK	p38	PW66, UM101, p38 inhibitors
	JNK	PW66, SP600125, JNK inhibitors
NALP3 inflammasome	NALP3 inflammasome	MCC950, parthenolide, glyburide, BHB, isoliquiritigenen
	IL-1β	Anakinra
Apoptosis	Apoptosis	antioxidants, zinc, apoptosis inhibitors
	caspases 3, 6, 7, 9	PW69, bithionol

**Table 3:** Small molecule anti-ricin inhibitors-mechanisms and targets.

Mechanism	Cell Target	Inhibitors
Receptor mimicry	RTB	Derivatives of glycosphingolipids, lactose and galactose
Endocytosis blockers	Early endosome	NaN3, cytochalasin D, colchicine
	Endosome	
Trafficking blockers	TGN	Retro-2, DA2MT, atorvastatin, brefeldin A, mansonone D
	ER	benzyl alcohol, 3′-Azido-3′-Deoxythimidine
Reductive activation inhibitors	PDI, TrxR, TMX,	auranofin, bacitracin
	glutathione disulfide oxidoreductase	
Active site and RTA inhibitors	Ribosomes	purine- pterin- and pyrimidine-based inhibitors, 4-fluorophenyl methyl 2-(furan-2-yl)quinolone-4-carboxylate, difluoromethylornithine, aptamers, RIP-α-sarcin/ricin loop interface blockers, baicalin
